# Formulation Development, Characterization and Antifungal Evaluation of Chitosan NPs for Topical Delivery of Voriconazole In Vitro and Ex Vivo

**DOI:** 10.3390/polym14010135

**Published:** 2021-12-30

**Authors:** Muhammad Khurshid Alam Shah, Abul Kalam Azad, Asif Nawaz, Shafi Ullah, Muhammad Shahid Latif, Habibur Rahman, Khalaf F. Alsharif, Khalid J. Alzahrani, Attalla F. El-Kott, Ashraf Albrakati, Mohamed M. Abdel-Daim

**Affiliations:** 1 Advanced Drug Delivery Lab, Gomal Center of Pharmaceutical Sciences, Faculty of Pharmacy, Gomal University, Dera Ismail Khan 29050, Pakistan; khursheed.peerzada@gmail.com (M.K.A.S.); asifnawaz676@gmail.com (A.N.); shafikustian@gmail.com (S.U.); 01drazad@gmail.com (M.S.L.); 2 Pharmaceutical Technology Unit, Faculty of Pharmacy, AIMST University, Bedong 08100, Kedah, Malaysia; 3 Department of Global Medical Science, Wonju College of Medicine, Yonsei University, Wonju 26426, Gangwon-do, Korea; pharmacisthabib@gmail.com; 4 Department of Clinical Laboratory Sciences, College of Applied Medical Sciences, Taif University, P.O. Box 11099, Taif 21944, Saudi Arabia; alsharif@tu.edu.sa (K.F.A.); ak.jamaan@tu.edu.sa (K.J.A.); 5 Biology Department, Faculty of Science, King Khalid University, Abha 61421, Saudi Arabia; elkottaf@kku.edu.sa; 6 Zoology Department, Faculty of Science, Damanhour University, Damanhour 22511, Egypt; 7 Department of Human Anatomy, College of Medicine, Taif University, P.O. Box 11099, Taif 21944, Saudi Arabia; a.albrakati@tu.edu.sa; 8 Pharmacology Department, Faculty of Veterinary Medicine, Suez Canal University, Ismailia 41522, Egypt

**Keywords:** polymers, PEG-4000, PG, topical delivery, voriconazole, chitosan

## Abstract

This study aims to develop chitosan-based voriconazole nanoparticles (NPs) using spray-drying technique. The effect of surfactants and polymers on the physicochemical properties, in vitro release, and permeation of NPs was investigated. The prepared NPs containing various surfactants and polymers (e.g., Tween 20 (T20), Tween 80 (T80), sodium lauryl sulfate (SLS), propylene glycol (PG), and Polyethylene glycol-4000 (PEG-4000)) were physiochemically evaluated for size, zeta potential, drug content, percent entrapment efficiency, in vitro release, and permeation across rats’ skin. A Franz diffusion cell was used for evaluating the in vitro release and permeation profile. The voriconazole-loaded NPs were investigated for antifungal activity against *Candida albicans* (*C. albicans*). The prepared NPs were in the nano range (i.e., 160–500 nm) and positively charged. Images taken by a scanning electron microscope showed that all prepared NPs were spherical and smooth. The drug content of NPs ranged from 75% to 90%. Nanoparticle formulations exhibited a good in vitro release profile and transport voriconazole across the rat’s skin in a slow control release manner. The NPs containing SLS, T80, and PG exhibited the best penetration and skin retention profile. In addition, the formulation exhibited a potential antifungal effect against *C. albicans*. It was concluded that the development of chitosan NPs has a great potential for the topical delivery of voriconazole against fungal infection.

## 1. Introduction

Fungal infections are one of the major healthcare problems worldwide. Superficial fungal infections of the hair, skin, and nails form the most numerous and widespread group of all mycoses [1]. They are most often caused by dermatophytes and yeasts. Although superficial fungal infections tend less to systemic infection, their treatment is increasingly difficult in high-risk patient groups such as AIDS patients, organ transplant recipients, and cancer patients undergoing immunosuppressive chemotherapy [2]. Azole antifungal agents are the most used antifungals in the clinical treatment of both superficial and systemic fungal infections [3]. Voriconazole (log P: 1.8) is a second-generation triazole available in both intravenous and oral formulations [4]. It has a wide spectrum against Candida spp., Aspergillus spp., and Cryptococcus neoformans, including those resistant to other commonly used antifungal agents. However, the oral administration of voriconazole is compromised by complicated pharmacokinetics, notable drug interactions, and relatively significant adverse events [5]. Furthermore, its intravenous administration is reported to cause heart rhythm problems [6]. Therefore, a need exists for a topical delivery system to overcome the limitations and enhance the antifungal effect of voriconazole against cutaneous candidiasis [7]. 

For the development of a novel drug delivery system, significant attention has been offered over the past two decades. The main aim of this study is to develop a novel topical drug delivery matrix to improve the therapeutic safety and efficacy of voriconazole-loaded NPs by altering their pharmacodynamic and pharmacokinetic parameters. Among different drug delivery systems, colloidal systems have a good reputation. The main colloidal drug delivery system is composed of polymeric NPs. This system is being investigated principally for being a controlled site-specific drug and for the improvement of bioavailability of poor water-soluble drugs [8]. Nano-carriers can be efficiently used as a mean of delivery for recently available biological active compounds [9]. For increasing the percutaneous absorption of the pharmaceutical formulations, nano-sized colloidal particles that act as a carrier have been developed, such as micelles, liposomes, inorganic NPs, and polymeric NPs [10]. The NPs have the capability to cross barriers of cells and tissues and have an improved reactive range, making them a suitable substance for biomedical applications [11]. By decreasing the size, the surface area increases; thus, smaller particles are more reactive and have some important effects [12]. The dermal and transdermal route offers greater surface area and drug therapy. The administration and termination of the drug over the skin facilitates both in the management of systemic and topical disorders [13]. The topical administration of a drug is a painless technique and a perfect route for the management of those disorders that require long-term therapy, e.g., chronic pain [14]. Hepatic first-pass metabolism can also be prevented [15]. 

Surfactants that reduce surface tension are used as enhancers for percutaneous absorption of the drug. They denature the keratin by swelling and disturbing the stratum corneum lipid layer for enhanced penetration of the drug across the skin [16]. The polar route causes changes in the protein structure and hydration of the skin. The nonpolar route changes the structure and composition of the lipid layers while the fatty acid enhancer flows easily across the lipid layers of the stratum corneum [17]. Chemical permeation enhancers speed up the absorption of the drug superficially applied to the skin [18]. 

Chitosan is the deacetylated by-product of chitin [19]. Chitosan has drawn significant attention from materials scientists because of its remarkable biological properties and commercial use [20]. It is nontoxic, has antimicrobial properties, and is biodegradable and biocompatible [21]. Voriconazole belongs to the azole group and is used to treat various fungal infections caused by various types of fungi [22]. The administration of voriconazole through IV route can cause irregular heart rhythm. To prevent such life-threatening consequences, voriconazole is applied topically to treat fungal infections [23]. Further investigation is necessary in terms of the toxicity and safety of this formulation for clinical trial and large-scale production. In this case, the GoNanoBioMat Safe-by-Design (SbD) approach is the best choice for further research because it provides all relevant steps for developing polymeric nano-biomaterials (NBMs) and the methodology and endpoints to test human health and environmental risks [24]. In the present study, chitosan-based NPs were prepared using spray-drying technique. Moreover, the main mechanism of the surfactants is to reduce the viscosity and surface tension of the chitosan-drug solution, resulting in smaller droplet production which then ultimately produces smaller NPs. Finally, the formulated NPs were characterized by determining the particle size, zeta potential, percentage of encapsulation efficiency (%EE), percentage of drug content (%DC), Fourier-transform infrared spectroscopy (FTIR), scanning electron microscope (SEM), in vitro drug release, ex vivo permeation study, skin retention analysis, and in vitro antifungal activity for the topical delivery of voriconazole.

## 2. Materials and Methods

### 2.1. Materials

Voriconazole was supplied by Laboratory Chemical Centre (Laboratory Chemical Centre, Lahore, Punjab 54000, Pakistan). Chitosan Low average molecular weight chitosan (100,000 g/mol) with degree of deacetylation of 75–85% and viscosity of 20–300 cP, tween 80 (T80), tween 20 (T20), acetic acid, sodium lauryl sulfate (SLS), propylene glycol (PG), and Polyethylene glycol-4000 (PEG-4000) were purchased from (Merck KGaA, 64293 Darmstadt, Germany). In addition, coconut oil was supplied by Hamdard Laboratories Pvt. Ltd. (67 Circular Road, Lahore, Punjab 54000, Pakistan). All the chemicals were of analytical grade.

### 2.2. Preparation of NPs

The formulation composition has described in detail as Table 1. Spray drier was used for the preparation of NPs. A solution of 0.25% *w/w* chitosan was dissolved in 1% acetic acid, with 1% *w/w* drug added to this solution and stirred continuously by a magnetic stirrer. Different surfactants and polymers were added to this solution with continuous stirring. Upon complete dissolution, the prepared solution was spray dried with the help of a spray drier (Pilotec YC-015, Shangai, China). The inlet temperature was kept at 70 °C and outlet temperature at 40 °C, with the chitosan solution containing the drug and surfactant fed at the rate of 10 mL/min. The simultaneous rate of air flow was 100 to 119 I/h and internal chamber vacuum pressure was kept at 06 bar, with spray mesh having a pore size of 5 µm used. The actuator vibration frequency was kept at 60 kHz. In the collecting chamber, the spray dried powder was collected. With the help of a rubber spatula, the collected powder was taken into an amber diagnostic vial. The collected sample was kept in a desiccator until required [25].

### 2.3. Characterization of NPs

#### 2.3.1. Particle Size and Zeta Potential

Photon correlation spectroscopy was used to determine the particle size and zeta potential of NPs (Malvern Zetasizer Nano series Nano-S and Nano-Z, Malvern Instruments Ltd., Worcestershire, UK). For analysis, 5 mg of NPs were dispersed in 5 mL of deionized water and analyzed on Zetasizer. Special tubes were used for zeta potential [26,27]. All experiments were performed in triplicate.

#### 2.3.2. Percentage of Drug Entrapment Efficiency (%EE) Determination

A fixed amount (15 mg) of NPs were taken and suspended in 30 mL of phosphate buffer pH 7.4. The solution was then centrifuged at 5000 rpm for 45 min. The supernatant was taken and analyzed on UV visible spectrophotometer (Shimadzu 1800, Nakagyo-Ku, Kyoto 604-8511, Japan) at wavelength 256 nm. The prepared NPs’ drug content and percent entrapment efficiency was calculated using Equation (1):

(1) $$\%\mathrm{EE}=\frac{\mathrm{Weight}\;\mathrm{of}\;\mathrm{drug}\;\mathrm{in}\;\mathrm{NPs}}{\mathrm{Weight}\;\mathrm{of}\;\mathrm{NPs}}\times 100$$

#### 2.3.3. Percentage of Drug Content (%DC) Determination

The collected pellets were then dissolved in 1% *v/v* acetic acid solution and analyzed by using UV visible spectrophotometer (Shimadzu 1800, Nakagyo-Ku, Kyoto 604-8511, Japan) at wavelength 256 nm [28]. The percentage of prepared NPs’ drug content was calculated using Equation (2):

(2) $$\%\mathrm{DC}=\frac{\mathrm{Amount}\;\mathrm{of}\;\mathrm{drug}\;\mathrm{in}\;\mathrm{nanoparticles}}{\mathrm{Amount}\;\mathrm{of}\;\mathrm{drug}\;\mathrm{used}\;\mathrm{in}\;\mathrm{formulation}}\times 100$$

#### 2.3.4. FTIR Analysis

At the end of the in vitro penetration study, the rat’s skin epidermis and dermis, including blank skin, formulation treated skin (VRZNA, VRZNB, VRZNC, VRZND, VRZNE, VRZNF, VRZNG), and pure drug treated skin, was investigated by ATR-FTIR spectroscopy. The ATR-FTIR spectra were taken in the frequency range of 4000–400 cm^−1^ with a spectral resolution of 4 cm^−1^ and with ambient air as a background using an FTIR spectrometer (PerkinElmer Inc., Waltham, MA, USA). The internal reflection element was a ZnSe crystal with a trapezoidal cut at 45 °C. The peak positions were determined using Perkin Elmer Spectrum Version 6.0.2 software (PerkinElmer, Waltham, MA, USA) [1]. 

#### 2.3.5. Surface Morphology

Chitosan NPs surface morphology was performed by using scanning electron microscopy (Carl Zeiss, GmbH, 73447 Oberkochen, Germany). The chitosan NPs were linked onto the studs using carbon tape. The prepared studs were analyzed directly at an increasing voltage of 15 kV under the scanning electron microscope. Chitosan NPs morphological images were taken at 20× level and images of the specific portion were captured. 

### 2.4. In Vitro Release

A Franz diffusion cell was used for in vitro release of the drug from the NPs [29]. An exact amount of 15 mg NPs of each formulation prepared by spray dryer was subjected to the donor compartment over the membrane (cellulose acetate membrane with pore size 0.20 µm) in the form of dry powder. Then, 2 mL of the sample was taken from the receptor compartment and 2 mL of fresh phosphate buffer (USP) solution of pH 5.5 was added to the recipient compartment. Samples were taken at intervals of 0 min, 30 min, 1 h, 2 h, 4 h, 8 h, 12 h, 16 h, 20 h, 24 h, and 28 h, and were analyzed on UV visible spectrophotometer (Shimadzu 1800, Nakagyo-Ku, Kyoto 604-8511, Japan) at 256 nm [30,31]. The temperature of the Franz diffusion cell was kept at 32 ± 1 °C. The same procedure for all the formulations was repeated in triplicate and the results were averaged. The Franz diffusion cell used had 6 mL capacity of the receptor compartment.

### 2.5. Skin Permeation and Retention

#### 2.5.1. Skin Preparation

For determining the skin permeations, excised skin of healthy male Sprague Dawley rats was used. The animal study protocol was revised and approved by the institutional animal care and research unit (204/QEC/GU, Ethical Review Board, Gomal University, Dera Ismail Khan 29050, Pakistan). The rats’ weights were 200–250 g and their ages were 3–4 months. The cervical dislocation method was used for the scarification of rats. With the help of a sharp blade, the rats’ ventral regions were shaved, and their skin surgically removed. The lipid layer attached with the removed skin was also detached with the help of the surgical blade and was washed with normal saline (0.9%) solution. The washed skin was then wrapped in aluminum foil and kept in a freezer at a temperature of −20 °C for further use [32].

#### 2.5.2. Skin Permeation Analysis 

To determine the skin permeation profile, the recipient chamber of the Franz diffusion chamber was filled with phosphate buffer (USP) with pH 7.4 due to the receptor chamber and medium having adequate solubility for the compound under study. This ensures that sink conditions are sustained throughout the length of the study, allowing the rate of absorption to proceed as it would normally under in vivo conditions with a functioning circulatory system. For water–soluble compounds, isotonic saline or buffered isotonic saline (pH~7.4) are considered the rational choice for the maintenance of a physiological environment [33]. Net 15 mg of NPs were taken and applied on the skin in the form of dry powder. The total amount of active drug in each formulation was 8.108 mg. The temperature of the cells was sustained at 37 ± 1 °C. A fixed volume (2 mL) of sample was collected from the recipient chamber at a specific interval of time and analyzed on UV visible spectrophotometer (Shimadzu 1800, Nakagyo-Ku, Kyoto 604-8511, Japan).

#### 2.5.3. Skin Retention Analysis

After the skin permeation study, the skin from the Franz diffusion cell was removed and softly rinsed with phosphate buffer saline pH 7.4. The washed skin was then cut into pieces that were placed in a beaker containing 20 mL of fresh phosphate buffer pH 7.4. This was then stirred with the help of a magnetic stirrer for 3 h. Then 5 mL of methanol was added to extract the drug and it was stirred again for 1 h. After that, 3 mL of the stirred solution was collected, filtered, and analyzed on UV-visible spectrophotometer (Shimadzu 1800, Nakagyo-Ku, Kyoto, 604-8511, Japan) [33].

### 2.6. In Vitro Antifungal Activity

The cup-plate technique was used to evaluate the antifungal activity of different formulations. Sabouraud dextrose agar media, comprised of dextrose 4 g, agar 1.5 g, and mycological peptone 1 g, was used. 

The sabouraud dextrose agar (5.5 g) was dissolved in 100 mL of distilled water with continuous heating and agitation. To dissolve the whole powder, the solution was boiled for 1 min with the medium maintained at pH 5.5. The prepared media was autoclaved at a temperature of 121 °C for 15 min.

Three sterile glass petri dishes of 93 mm were taken, with the media poured into the dishes aseptically. The plates were kept for solidification. When the agar media solidified, a sterile cork borer was used to pierce the surface of the agar plate. The *C. albicans* were inoculated by streaking of the surface of the agar media. Small holes of 8mm were formed in all three petri dishes, with 6 µg of voriconazole 1% *w/w* NPs taken and placed in the first dish. In the second dish, 6 µg of chitosan NPs (0.25% *w/w*) were placed, while in the third dish, 6 µg of chitosan based voriconazole NPs were subjected to holes. After subjecting the three samples, the petri dishes were kept and left for at least 30 min and were incubated for 24 h at a temperature of 25 ± 1 °C. After 24 h, the most uniform outer diameter of the zone of inhibition was recorded in millimeters using a vernier caliper (VWR International Inc., Radnor, PA, USA) and expressed in radial dimensions. The diameter of zones, including the diameter of the well, was recorded. Each assay was carried out in triplicate [34].

### 2.7. Statistical Analysis

The data was analyzed using Statistical Package Minitab^®^ version 20 (Minitab, LLC, Pennsylvania, State College, PA 16801, USA) and reported as a Kolmogorov-Smirnov (K-S) test before applying one-way ANOVA. It was statistically analyzed using ANOVA (one-way analysis of variance), with post hoc analysis by Tukey HSD test (IBM^®^ SPSS^®^ Statistics version 19, Armonk, New York, NY 10504-1722, USA). Results were considered statistically significant with a value of *p* ˂ 0.05. All tested data were described in triplicate (n = 3) and mean ± standard deviation (S.D.).

## 3. Results and Discussions

### 3.1. Physicochemical Characterization of NPs

#### 3.1.1. Size and Zeta Potential

The results of the particle size of freshly prepared voriconazole-loaded NPs’ formulations are shown in Table 2.

The particle size of formulations ranged from 167 nm to 475 nm. The lowest size of NPs was obtained from the formulations VRZNB (167 ± 8.23 nm), followed by VRZND (189 ± 7.34 nm), VRZNE (202 ± 9.12 nm), and VRZNC (210 ± 9.11 nm), which is an ideal size for the transdermal or topical drug delivery system. The introduction of surfactants reduced the size of the NPs. This might be due to the reduction in surface tension of the polymer drug solution, resulting in smaller droplets and hence small particles being produced. Regarding particle size, as a drug delivery system, the submicron-sized NPs have a number of advantages over the microparticles [35]. For penetration to lower layers of the human skin, particles of 500–1000 nm can penetrate easily while smaller particles can penetrate the deeper layers of the skin [36]. On the other hand, if particle size is increased the drug will diffuse slowly [37]. Particle size also has an influence on polymer degradation [38]. 

In determining the surface charge and potential stability of the nanoparticle system, zeta potential is an essential characterization process. Usually, for the stability of colloidal dispersion, very large negative or positive values of zeta potential are desired, as electrostatic repulsion results in the non-aggregation of particles having the same charge. Because of the existence of chitosan, all the formulations showed positive zeta potential values. The zeta potential of formulation VRZNA to VRZNG ranged from 38 mV to 45 mV. The zeta potential of the prepared nanoparticle formulations is shown in Table 2. The chitosan–voriconazole NPs without surfactants have zeta potential of 45 ± 3.1 mV. The introduction of surfactants slightly reduced the positive charge of prepared NPs, as shown in Table 2. Zeta potential can be affected by several factors influencing particle surface, such as surfactant makeup, distribution, and dispersal of drug into the NPs [39], and post-production developments (for example, purification and freeze drying [40]). The adjacent pH and the chemical configuration rule the type of charge and the zeta potential magnitude of a nanocarrier. By increasing the number of cationic excipients and lowering the surrounding pH, the extent of zeta potential usually increases, with positively charged zeta potential created by cationic constituents. Zeta potentials support NPs more when drugs enter through the transdermal route than into the vesicular system.

Conversely, powerful charge–charge repulsion may occur when excessive amount of negative charge is present on a nanoparticle surface which blocks the penetration into dense layers of the skin. Polysaccharide NPs are available as both negatively charged and positively charged drug carriers. Both the positively charged and negatively charged drug carriers have a high tendency to transport the drug through a transdermal route in contrast to neutral particles [41]. The cationic polysaccharide NPs interact electrostatically with the negatively charged lipids and keratin of stratum corneum. They can melt down the epidermis and raises transdermal drug diffusion similar to a negatively charged carrier [42]. 

#### 3.1.2. Entrapment Efficiency (%EE)

The resultant percent entrapment efficiency of the NPs, ranging from 38.11% to 58.48%, was found to be acceptable and is shown in Table 2. The minimum entrapment efficiency was 38.11% and the maximum entrapment efficiency was 58.48%. The presence of surfactants improves the %EE, as shown in Table 2. This might be due to the low surface tension of polymer solution, resulting in more flexible droplet production and hence a high percentage entrapment of the drug. High entrapment efficiency can be obtained if drug leaking does not occur from the matrix during preparation of the formulation. Drug loading is dependent on several factors related to the drug, excipients, and preparation method. Preparation of pharmaceutical formulations with high doses is supported by high drug loading, thus decreasing the substance of the excipients, hence the biocompatibility profile improves [43]. When encapsulation of a slightly water-soluble drug is required, it is recommended that the temperature during the manufacturing procedure is kept lower, with the main objective of reducing the drug solubility in water and preventing the partitioning of the drug into the aqueous phase. Hydrophilic drugs are converted to fat-soluble lipid drugs using a conjugate method [44]. 

#### 3.1.3. Drug Content (%DC)

All formulations were analyzed for their drug content spectrophotometrically, shown in Table 2. Of the formulations, VRZND showed the highest drug content. The drug content for the voriconazole-loaded chitosan NPs fluctuated from 63.22% to 91.40%. As the particle size increases, the extent of diffusional route into aqueous phase also increases. Thus, the loss of the drug through diffusion process is decreased, resulting in elevated drug content. When the concentration of the polymer increases, the precipitation time required for the polymers decreases. Hence, the drug molecule cannot diffuse out in short time from the NPs, resulting in increased drug content [45]. This result indicates that there was no drug loss by manufacturing process or by excipients used in the formulation.

#### 3.1.4. FTIR Analysis

**Figure 1:**
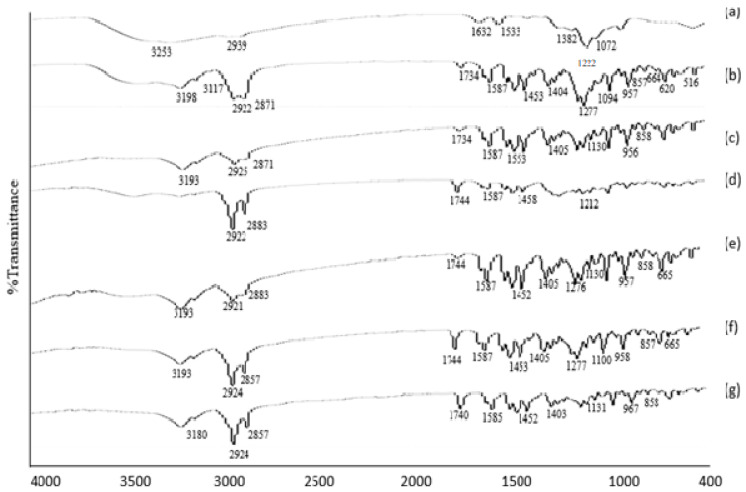
FTIR spectra of (**a**) VRZNA, (**b**) VRZNB, (**c**) VRZNC, (**d**) VRZND, (**e**) VRZNE, (**f**) VRZNF, and (**g**) VRZNG.

Voriconazole is a composite component comprised of active constituent (2R,3S)-2-(2,4-difluorophenyl)-3-(5-fluoro-4-pyrimidinyl)-1-(1H-1,2,4-triazol-1-yl)-2-butanol. In FTIR, the peak spectra of absorption for voriconazole were obtained in the region of 3136 cm^−1^_._ Peaks between 3011–2022 cm^−1^ relating to the stretching vibrations of OH.1533 cm^−1^ were consigned to the alkane CH, C = C aromatic, and aryl C–N stretches, respectively. The observed spectral region 1000–1200 cm^−1^ represents the characteristic vibration multiple bands for C–F. FTIR spectra of (a) VRZNA, (b) VRZNB, (c) VRZNC, (d) VRZND, (e) VRZNE, (f) VRZNF, and (g) VRZNG. All of the spectra are illustrated in Figure 1. The characteristic peak was present, and no new peaks appeared during FTIR analysis. There was no significant interaction observed between the drug and the excipient. These results indicate that voriconazole is compatible with chitosan and other excipients of the formulation.

#### 3.1.5. Surface Morphology 

**Figure 2:**
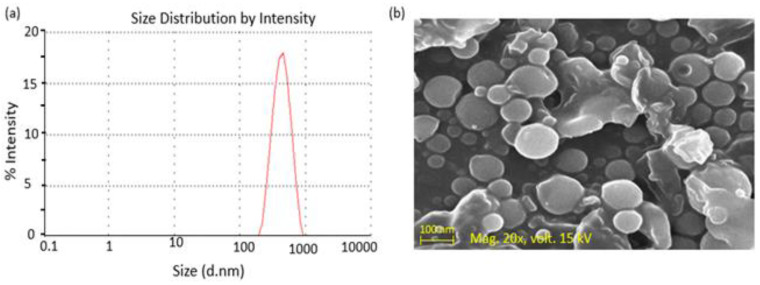
Particle size distribution (**a**) and SEM morphology of chitosan NPs containing voriconazole of formulation VRZNC (**b**).

The particle size distribution curve is presented at Figure 2a. SEM images showed that most of the NPs formed were spherical in shape and had a smooth surface, as shown in Figure 2b. For particle size distribution and SEM morphology analysis, chitosan NPs containing voriconazole formulation (VRZNC) were selected based on a maximum release (94.19%) at 1680 min compared to other formulations (*p* < 0.05).

### 3.2. In Vitro Release

**Figure 3:**
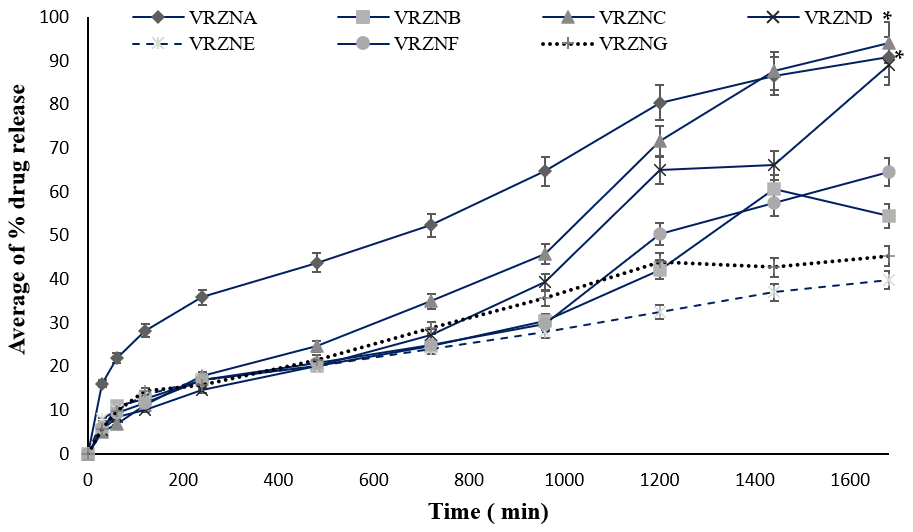
In vitro average of % drug release of formulations: n = 3, mean ± S.D., * *p* < 0.05 considered statistically significantly different from VRZNB, VRZND, VRZNE, VRZNF, and VRZNG.

The average percent release of the different formulations having different surfactants and polymers ranged from 39% to 94%, as shown in Figure 3. The formulations VRZNA and VRZNC showed maximum release at 1680 min (90.87% and 94.19%), which is significantly different (*p* < 0.05) from formulations VRZNB, VRZND, VRZNE, VRZNF, and VRZNG. Here, formulation VRZNC showed maximum percent release due to the combination of coconut oil and T80. This may have had a synergy effect on the release behavior, as this combination was not employed for the rest of the formulations in the current study. There are numerous methods that control the release of the drug from the chitosan NPs, e.g., polymer swelling, drug diffusion across the polymeric medium, diffusion of the adsorbed drug, polymer attrition and/or degradation. In general, swelling initiates once the polymer meets the surrounding dissolution medium. Therefore, the initial burst release from the chitosan NPs is either because of diffusion of the drug from the surface of the polymer or swelling of the polymer, forming apertures. Sometimes, subsequent physical erosion may occur due to breakage of bonds when polymer degradation occurs. This might be explained by the fact that smaller particle sizes increase the surface area of the matrix which increases the rate of osmosis and diffusion of NPs. Moreover, in the case of the microemulsion technique, the entrapment efficiency was lower which ultimately affected the drug release. The polymer concentration produced a more sustained increase in drug release [46]. 

Polymer erosion at a uniform rate across the medium is known as homogenous erosion, while erosion from the surface towards the inner core of the polymer is called heterogeneous erosion [47]. Drug distribution in the matrix is one of the most prominent factors affecting NPs’ drug release. The drug can be dispersed homogenously or nonhomogenously within every nanoparticle, depending on the composition of the particle and method of producing the nanoparticle. In contrast, each nanoparticle zone is formed with enough drug in it [38]. The perfect conditions in the first case resulted in a sustained release over time when the drug was slowly released from NPs. In the second case, the liberation of the drug was a rapid “burst release” because it deposited in the outer layers of NPs [48]. 

### 3.3. Skin Permeation

**Figure 4:**
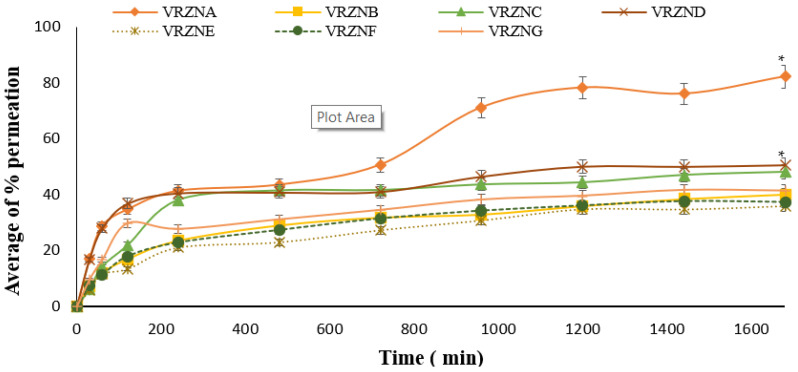
In vitro average of % permeation of formulations: n = 3, mean ± S.D., * *p* < 0.05 considered statistically significantly different from VRZNB, VRZNC, VRZNE, VRZNF, and VRZNG.

Compared to the permeation of pure drug which was 9.75%, all formulations having different surfactants exhibited a good percentage of in vitro permeation, as shown in Figure 4. The in vitro permeation parameters were calculated by plotting a graph showing the quantity of drug permeated vs. time. Various factors were involved, such as the concentration of surfactant, contact time, exposure type, and the individual response. When the surfactants bind with the protein leading to its denaturation, this results in swelling of the stratum corneum. The solubilization of fluid lipids and the abstraction of calcium or other multivalent ions to reduce corneocyte adhesion enhance the accessibility of the proteins in the lower regions of the stratum corneum [49]. Isolated stratum corneum swelling was first investigated by Rhein et al. A powerful hydrophobic interaction is encouraged between protein and surfactant molecules when the cationic site of the protein interacts with the anionic head groups. This results in the ultimate denaturation of the protein molecules. Anionic surfactants accelerate the protein dissolution out of the skin, with protein sulfhydryl groups released from the sclera. Final denaturization is achieved by the reaction of various skin enzymes. Protein binds with nonionic surfactants through weak hydrophobic linkage. Skin barrier function is modified by the integration of surfactants into the lipid bilayer causing denaturization. Some important changes in lipid composition of the stratum corneum caused by sodium dodecyl sulfate have been studied by Fulmer et al. Permeability may be improved by the emulsification of lipids of the stratum corneum by using surfactants of low concentration. It was observed that SLS increases the epidermal lipid fluidity, and hence penetration into the skin is enhanced. Monomers of surfactant can contact the living segment of the epidermis and integrate with keratinocytes once the lipid barrier of the skin has been discomposed/weakened. It was observed that the treatment of skin with SLS (0.6% *v/v*) for a short period put the stratum corneum out of order and did not result in thickening of the epidermis [50].

SLS has the capability to change the structural organization of the skin lipids and show the same effect when it is used above the critical micellar concentration. The lipophilicity values of those compounds, which are less than the optimum values for SLS, have the capability to enhance their penetration rate [51]. Polysorbate 80 and 20 are recognized to increase the permeability of phospholipid membranes, causing leakage of low molecular mass compounds. Polysorbate 20 and 80 also produce variation in the physicochemical characteristics of biomembranes enhancing the permeability of sarcoplasmic reticulum. It is thought that nonionic surfactants, as penetration enhancers, seep into the skin’s intercellular lipid bilayers; thus, crystallinity of these lipid bilayers is reduced, and their permeability features are intensified. It is clear from the permeability data that T80 used in this study as a permeation enhancer increased permeation by decreasing skin resistance to the diffusion of voriconazole through hairless rat skin in vitro.

The following mechanism explains the speedy penetration of the drug across the skin membrane in the presence of T80. Initially, the drug molecule once absorbed into the skin surface results in a gradient of the drug’s high thermodynamic activity. This is what makes it an effective force for subsequent drug permeation. The second effect of the permeation enhancers is that they reduce obstructive characteristics of the stratum corneum. Third, by changing or modifying the stratum corneum structure, the intracellular lipid barrier becomes looser and thus more permeable to permeation enhancers [52]. Polyethylene glycols show weak skin penetration due to steric limitations proposed by adsorbed water molecules. Conversely, skin with compromised barrier and burns (>20%) enhances polyethylene glycol penetration regardless of molecular weight. Despite this, polyethylene glycol does not irritate the skin. Polyethylene glycol used in low quantity has a great impact on the barrier structure of the skin by lowering the surface tension and conditioning the stratum corneum. Solid polyethylene glycol is typically used in topical preparations according to its consistency and is used extensively in cosmetics. By increasing the molecular weight of polyethylene glycol, the release of drugs from NPs is decreased, hence permeation is also decreased. Release modifiers, substantially surrounded drugs in polyethylene glycol matrix, are released either by diffusion or erosion-controlled method or a combination of both. Water permeates from the shell to the inner core and dissolves the drug followed by diffusion. The drug is so strongly bonded by chemical linkage that the water does not solubilize it when permeated in this system. Sustained release of the drug occurs because of the mechanism followed by PEG-3000 to PEG-5000, called erosion-controlled drug release mechanism. When the drug is linked with polyethylene glycol, its release depends on the type of linkage and whether it is releasable or permanent. The half-life and water solubility are enhanced and cause steric interference by blocking the bioactivity at the site of action when PEGylation is performed with high molecular weight polyethylene glycol. Prodrugs and releasable polyethylene glycol are the same, wherein the biological setting PEGylation of active conjugate through hydrolysable linkers would release its active conjugate upon chemical, enzymatic, and thermal stimulation [52]. Among the results obtained from different formulations, the formulation VRZNA containing T80 showed the highest percent permeation value (82.21%) at 1680 min (*p* < 0.05). VRZNC containing T80 and coconut oil as permeation enhancers exhibited permeation up to 48.11%; VRZND containing surfactant sodium lauryl sulfate and coconut oil as permeation enhancers exhibited permeation values up to 50.63%; and VRZNG containing PG had average percent permeation up to 41.39% at 1680 min.

### 3.4. Drug Retention

**Figure 5:**
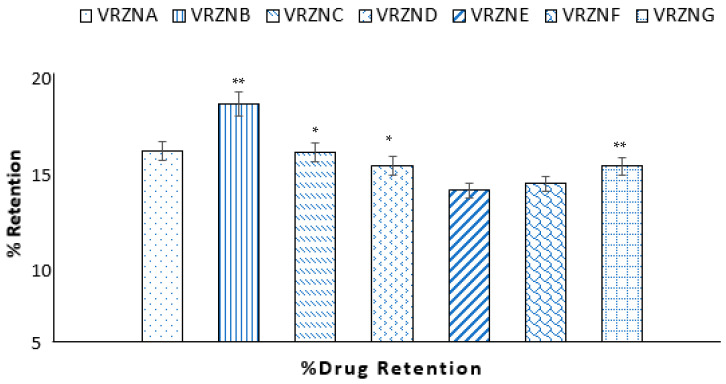
**%** Drug retention of different formulations: n = 3, mean ± S.D., * *p* < 0.05 and ** *p* < 0.001 considered statistically significantly different from VRZNA, VRZNE, and VRZNF.

The drug retention of different formulations is shown in Figure 5. The skin drug retention of different formulations ranges from 11.90 ± 0.99% to 18.72 ± 1.31%. The formulations VRZNA and VRZNB show higher percent drug retention (*p* < 0.05). Skin presents significant evidence regarding the concentration of the active drug that can reach the site of action and about possible side effects due to systemic exposure. The variability in data from low to high suggests that the main source is skin and the variation in its thickness/weight. The results also show low retention in the stratum corneum despite the lipophilic nature of the drug. This might be due to the low solubility in the stratum corneum lipids [53]. Another reason for the low stratum corneum retention might be the penetration pathway, which is not forcing diffusion across the skin layers but might be via skin appendages [54]. 

### 3.5. Analysis of Skin Epidermis and Dermis by ATR-FTIR

**Figure 6:**
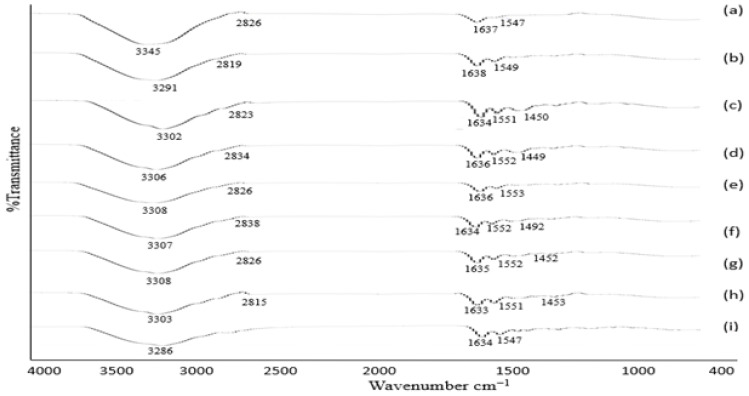
ATR-FTIR of skin epidermis: (**a**) blank skin, (**b**) skin treated with formulation VRZNA, (**c**) skin treated with formulation VRZNB, (**d**) skin treated with formulation VRZNC, (**e**) skin treated with formulation VRZND, (**f**) skin treated with formulation VRZNE, (**g**) skin treated with formulation VRZNF, (**h**) skin treated with formulation VRZNG, and (**i**) skin treated with pure drug.

**Figure 7:**
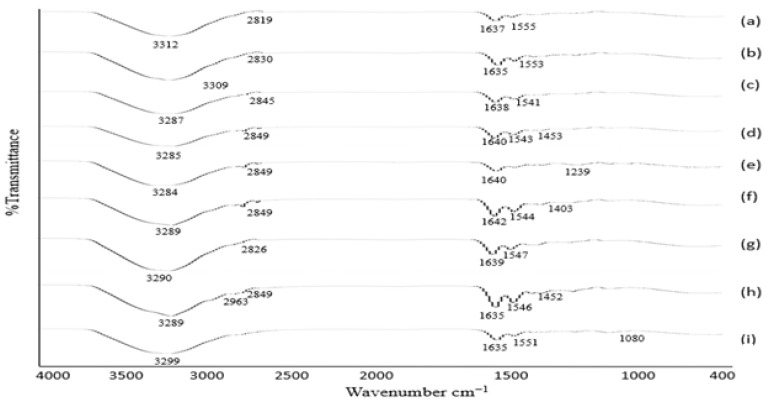
ATR-FTIR of skin dermis: (**a**) blank skin, (**b**) skin treated with formulation VRZNA, (**c**) skin treated with formulation VRZNB, (**d**) skin treated with formulation VRZNC, (**e**) skin treated with formulation VRZND, (**f**) skin treated with formulation VRZNE, (**g**) skin treated with formulation VRZNF, (**h**) skin treated with formulation VRZNG, and (**i**) skin treated with pure drug.

The ATR-FTIR results of skin epidermis and dermis are shown in Figure 6 and Figure 7. The wavenumbers of peaks related to C–H 2917–2963 cm^−1^, 2819–2849 cm^−1^, and 2815–2838 cm^−1^ portions of untreated and treated epidermis and dermis. In addition, the wavenumber of the FTIR peak of untreated epidermis and dermis increased in skin treated with NPs 2819–2849 cm^−1^ and 2815–2838 cm^−1^. Transdermal drug diffusion was supported by the collective skin fluidization effects. FTIR peak 2849 cm^−1^ was responsible for the skin fluidization. The wavenumbers of FTIR peaks represent O–H/N–H 3286–3308 cm^−1^ bonds or ceramides, keratins, or lipids, while the peak from 1633–1638 cm^−1^ of the epidermis [55] and from 1635–1642 cm^−1^ characterizes secondary amine N–H bend and C=O/C–N. The 1547–1553 cm^−1^ parts of the epidermis managed to have smaller wavenumbers with skin treated by NPs. However, the wavenumbers of FTIR peaks representing the O–H/N–H 3284–3309 cm^−1^ portion of the dermis tended to have smaller wavenumbers, but the peaks allocated to C=O/C–N 1541–1555 cm^−1^ showed high wavenumbers with skin treated by NPs. The hydrophobic organization bound to the cellular domain of the skin was fluidized by NPs. These results follow the previous study [55]. The hydrophilic regime of the epidermis O–H/N–H, C=O/C–N and dermis O–H/N–H experienced defluidization, with dermal C=O/C–N portions receiving fluidization by the same treatment.

Detailed insights into the organization of the stratum corneum can be gained through the study of the vibrations of amide, amine, and carboxylic groups and the frequencies of the methylene stretching, scissoring, and rocking vibrations. Therefore, it is used to study the lateral lipid organization of the intercellular lipid matrix in stratum corneum, which is essential for the barrier function of stratum corneum, as more densely organized membranes are less permeable to substances. The stretching vibrations are used to determine whether lipids are in an ordered (hexagonal or orthorhombic lateral packing) or disordered packing (liquid phase) [56]. It can be concluded that treatment with microemulsion contributes to an increase in the fluidity of stratum corneum lipids, which enhances skin penetration [1]. The disordering of the stratum corneum intercellular lipids after microemulsion treatment can be attributed to the membrane permeability enhancing effect of oleic acid. Oleic acid is well known for its ability to interact with the lipids in the stratum corneum, leading to a temporary and reversible increase in their fluidity, such that drug mobility is also increased.

### 3.6. In Vitro Antifungal Activity

**Figure 8:**
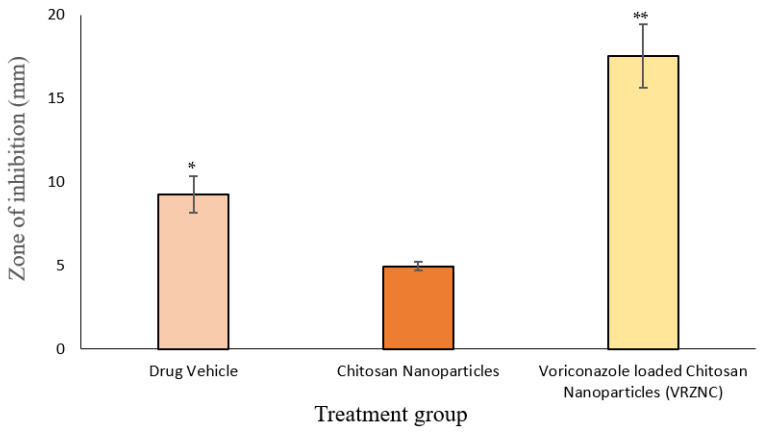
In vitro antifungal activity of drug vehicle, chitosan NPs, and voriconazole-loaded chitosan NPs against *C. albicans*: n = 3, mean ± S.D., * *p* < 0.05 and ** *p* < 0.001 considered statistically significant different from without drug-loaded chitosan nanoparticles.

The agar diffusion method was used for this microbiological study. Results showed that voriconazole NPs in combination with chitosan significantly inhibit the growth of *C. albicans*, when compared to simple voriconazole (1% *w/w*), and the antifungal activity is enhanced. The mean diameter of zones of inhibition against *C. albicans* were: voriconazole (1% *w/w*), 9.25 ± 1.35 mm; chitosan (0.25% *w/w*), 4.95 ± 1.45 mm; and chitosan-based voriconazole NPs, 17.55 ± 1.68 mm. The zones of inhibition for voriconazole NPs were higher and this is due to a synergistic effect of the drug and chitosan, shown in Figure 8. Shaimaa El-Housiny et al. reported fluconazole-loaded NPs’ formulations for topical delivery and found a potential therapeutic effect against antifungal infection [57].

## 4. Conclusions

In this study, chitosan-based voriconazole NPs were prepared with different surfactants and polymers. Moreover, their effects were also investigated based on physicochemical properties. The prepared voriconazole-loaded chitosan particles were from 167 nm to 475 nm, which are considered as NPs. ATR-FTIR results showed there was no interaction among the different excipients of the formulations. The drug content of NPs ranged from 75% to 91%. All the NPs’ formulations showed a valuable aptitude to deliver voriconazole through a rat’s skin. The NPs containing SLS, T80, and PG exhibited the best penetration profile. Skin drug retention was also increased by using different surfactants. It is also deduced from the ATR-FTIR results that NPs mainly affect the lipids and proteins of the stratum corneum, resulting in enhanced permeation and retention of the drug. A slight initial burst effect was observed, indicating that voriconazole was non homogeneously dispersed in the nanoparticle dispersion process and that no significant amount of drug was adsorbed onto the nanoparticle surface. NPs maintained prolonged drug level in the skin layer and the distribution of the drug from the chitosan-coated formulation up to 1680 min showed it was above the minimum inhibitory concentration level. Thus, it was obviously beneficial to kill the fungal spores that generally remain dormant and survive with conventional treatment and cause a recurrence of the disease. Therefore, voriconazole-loaded, chitosan-coated NPs are promising for the bioavailability improvement of effective transdermal delivery. It can be concluded that the development of NPs’ formulations has great potential for topical delivery against fungal infection.

## Figures and Tables

**Table 1:** Formulation composition (chitosan, voriconazole, coconut oil, T20, T80, SLS, PEG4000, and PG) of NPs.

F. Code	Drug % *w/w*	Chitosan % *w/w*	Coconut Oil % *w/w*	Surfactants and Polymers				
				T80 % *w/w*	SLS % *w/w*	T20 % *w/w*	PEG4000 % *w/w*	PG % *w/w*
VRZNA	1	0.5	0.25	-	-	-	-	-
VRZNB	1	0.5	-	0.6	-	-	-	-
VRZNC	1	0.5	0.25	0.6	-	-	-	-
VRZND	1	0.5	0.25	-	0.6	-	-	-
VRZNE	1	0.5	0.25	-	-	0.6	-	-
VRZNF	1	0.5	0.25	-	-	-	0.6	-
VRZNG	1	0.5	0.25	-	-	-	-	0.6

**Table 2:** Average particle size, zeta potential, % drug entrapment, and % drug content: n = 3, mean ± S.D., * *p* < 0.05 and ** *p* < 0.001 considered statistically significantly different.

F. Code	Average Size (nm)	Zeta Potential (mV)	% EE	% DC
VRZNA	475 ± 15.30	45 ± 3.11	38.11 ± 0.51	63.22 ± 0.58 **
VRZNB	167 ± 8.23 **	44 ± 2.70	58.48 ± 0.85 **	85.05 ± 0.63 *
VRZNC	210 ± 9.11 *	41 ± 1.92	41.49 ± 1.13	76.95 ± 0.50
VRZND	189 ± 7.34 *	39 ± 2.56	52.44 ± 0.35 *	91.40 ± 0.10 *
VRZNE	202 ± 9.12 *	40 ± 1.82	49.54 ± 0.41	91.02 ± 0.25 *
VRZNF	279 ± 11.41	42 ± 1.64	41.89 ± 0.39	75.02 ± 0.75
VRZNG	267 ± 10.17	41.5 ± 2.91	51.71 ± 0.25 *	90.52 ± 0.39 *

## Data Availability

Data will be available upon request.

## References

[1] Qurt M.S., Esentürk İ., Tan S.B., Erdal M.S., Araman A., Güngör S. (2018). Voriconazole and sertaconazole loaded colloidal nano-carriers for enhanced skin deposition and improved topical fungal treatment. J. Drug Deliv. Sci. Technol..

[2] Kumar R., Sinha V.R. (2014). Preparation and optimization of voriconazole microemulsion for ocular delivery. Colloids Surf. B Biointerfaces.

[3] Latif M.S., Azad A.K., Nawaz A., Rashid S.A., Rahman M., Al Omar S.Y., Bungau S.G., Aleya L., Abdel-Daim M.M. (2021). Ethyl Cellulose and Hydroxypropyl Methyl Cellulose Blended Methotrexate-Loaded Transdermal Patches: In Vitro and Ex Vivo. Polymers.

[4] Zonios D.I., Bennett J.E. (2008). Update on azole antifungals. Semin. Respir. Crit. Care Med..

[5] El-Hadidy G.N., Ibrahim H.K., Mohamed M.I., El-Milligi M.F. (2012). Microemulsions as vehicles for topical administration of voriconazole: Formulation and in vitro evaluation. Drug Dev. Ind. Pharm..

[6] Raju Y.P., Hyndavi N., Harini Chowdary V., Nair R.S., Basha D.J., Tejeswari N. (2017). In vitro assessment of non-irritant microemulsified voriconazole hydrogel system. Artif. Cells Nanomed. Biotechnol..

[7] Sable C.A., Strohmeier K.M., Chodakewitz J.A. (2008). Advances in antifungal therapy. Annu. Rev. Med..

[8] Ghai R., Nagarajan K., Arora M., Grover P., Ali N., Kapoor G. (2020). Current strategies, and novel drug approaches for Alzheimer disease. CNS Neurol. Disord. Drug Targets.

[9] Bandopadhyay S., Manchanda S., Chandra A., Ali J., Deb P.K. (2020). Overview of different carrier systems for advanced drug delivery. Drug Delivery Systems.

[10] Wani T.U., Mohi-ud-Din R., Majeed A., Kawoosa S., Pottoo F.H. (2020). Skin permeation of NPs: Mechanisms involved and critical factors governing topical drug delivery. Curr. Pharm. Des..

[11] Sarma A., Bania R., Devi J.R., Deka S. (2021). Therapeutic nanostructures and nanotoxicity. J. Appl. Toxicol..

[12] Arenz M., Mayrhofer K.J., Stamenkovic V., Blizanac B.B., Tomoyuki T., Ross P.N., Markovic N.M. (2005). The effect of the particle size on the kinetics of CO electrooxidation on high surface area Pt catalysts. J. Am. Chem. Soc..

[13] Alexander A., Khichariya A., Gupta S., Patel R.J., Giri T.K., Tripathi D.K. (2013). Recent expansions in an emergent novel drug delivery technology: Emulgel. J. Control. Release.

[14] Seetharam A.A., Choudhry H., Bakhrebah M.A., Abdulaal W.H., Gupta M.S., Rizvi S.M.D., Moin A. (2020). Microneedles drug delivery systems for treatment of cancer: A recent update. Pharmaceutics.

[15] Sutradhar K.B., Amin M.L. (2013). Nanoemulsions: Increasing possibilities in drug delivery. Eur. J. Nanomed..

[16] Prokai L., Nguyen V., Jasti B.R., Ghosh T.K. (2021). Principles and applications of surface phenomena. Theory and Practice of Contemporary Pharmaceutics.

[17] Akhlaq M., Azad A.K., Fuloria S., Meenakshi D.U., Raza S., Safdar M., Nawaz A., Subramaniyan V., Sekar M., Sathasivam K.V. (2021). Fabrication of Tizanidine Loaded Patches Using Flaxseed Oil and Coriander Oil as a Penetration Enhancer for Transdermal Delivery. Polymers.

[18] Lin X., Wang Z., Ou H., Mitragotri S., Chen M. (2020). Correlations between skin barrier integrity and delivery of hydrophilic molecules in the presence of penetration enhancers. Pharm. Res..

[19] Seenuvasan M., Sarojini G., Dineshkumar M. (2020). Recovery of chitosan from natural biotic waste. Current Developments in Biotechnology and Bioengineering.

[20] Parhi R. (2020). Drug delivery applications of chitin and chitosan: A review. Environ. Chem. Lett..

[21] Anantrao J.H., Nath P.A., Nivrutti P.R. (2021). Drug penetration enhancement techniques in transdermal drug delivery system: A review. J. Pharm. Res. Int..

[22] Borchard G., Som C., Zinn M., Ostafe V., Borges O., Perale G., Wick P. (2020). Polymeric nano-biomaterials for medical applications: Advancements in developing and implementation considering safety-by-design concepts. Front. Bioeng. Biotechnol..

[23] Esentürk İ., Balkan T., Özhan G., Döşler S., Güngör S., Erdal M.S., Sarac A.S. (2020). Voriconazole incorporated nanofiber formulations for topical application: Preparation, characterization, and antifungal activity studies against Candida species. Pharm. Dev. Technol..

[24] Chachuli S.H.M., Nawaz A., Shah K., Naharudin I., Wong T.W. (2016). In vitro investigation of influences of chitosan NPs on fluorescein permeation into alveolar macrophages. Pharm. Res..

[25] Azad A.K., Al-Mahmood S.M.A., Chatterjee B., Wan Sulaiman W.M.A., Elsayed T.M., Doolaanea A.A. (2020). Encapsulation of black seed oil in alginate beads as a pH-sensitive carrier for intestine-targeted drug delivery: In vitro, in vivo and ex vivo study. Pharmaceutics.

[26] Bera H., Yasir F.A., Virendra G., Kok F.L., Pramod K., Prajakta T., Azad A.K., Dongmei C., Mingshi Y. (2020). Carboxymethyl fenugreek galactomannan-g-poly (N-isopropylacrylamide-co-N, N′-methylene-bis-acrylamide)-clay based pH/temperature-responsive nanocomposites as drug-carriers. Mater. Sci. Eng. C.

[27] Peltonen L., Aitta J., Hyvönen S., Karjalainen M., Hirvonen J. (2004). Improved entrapment efficiency of hydrophilic drug substance during nanoprecipitation of poly (I) lactide NPs. AAPS PharmSciTech.

[28] Azad A.K., Al-Mahmood S.M.A., Kennedy J.F., Chatterjee B., Bera H. (2021). Electro-hydrodynamic assisted synthesis of lecithin-stabilized peppermint oil-loaded alginate microbeads for intestinal drug delivery. Int. J. Biol. Macromol..

[29] Akhlaq M., Azad A.K., Ullah I., Nawaz A., Safdar M., Bhattacharya T., Uddin A.B., Abbas S.A., Mathews A., Kundu S.K. (2021). Methotrexate-loaded gelatin and polyvinyl alcohol (Gel/PVA) hydrogel as a pH-sensitive matrix. Polymers.

[30] Nawaz A., Wong T. (2016). Quantitative characterization of chitosan in the skin by Fourier-transform infrared spectroscopic imaging and ninhydrin assay: Application in transdermal sciences. J. Microsc..

[31] Khan T.A., Azad A.K., Fuloria S., Nawaz A., Subramaniyan V., Akhlaq M., Safdar M., Sathasivam K.V., Sekar M., Porwal O. (2021). Chitosan-Coated 5-Fluorouracil Incorporated Emulsions as Transdermal Drug Delivery Matrices. Polymers.

[32] Irfan M.M., Shah S.U., Khan I.U., Munir M.U., Khan N.R., Shah K.U., Mahmood S. (2021). Physicochemical characterization of finasteride nanosystem for enhanced topical delivery. Int. J. Nanomed..

[33] Finnin B., Walters K.A., Franz T.J. (2012). In vitro skin permeation methodology. Transdermal and Topical Drug Delivery: Principles and Practice.

[34] Chandrasekar V., Knabel S.J., Anantheswaran R.C. (2015). Modeling development of inhibition zones in an agar diffusion bioassay. Food Sci. Nutr..

[35] Redhead H.M., Davis S.S., Illum L. (2001). Drug delivery in poly (lactide-co-glycolide) NPs surface modified with poloxamer 407 and poloxamine 908: In vitro characterisation and in vivo evaluation. J. Control Release.

[36] Pal S.L., Jana U., Manna P.K., Mohanta G.P., Manavalan R. (2011). Nanoparticle: An overview of preparation and characterization. J. Appl. Pharm. Sci..

[37] Urbán-Morlán Z., Ganem-Rondero A., Melgoza-Contreras L.M., Escobar-Chávez J.J., Nava-Arzaluz M.G., Quintanar-Guerrero D. (2010). Preparation and characterization of solid lipid NPs containing cyclosporine by the emulsification-diffusion method. Int. J. Nanomed..

[38] Alvarez-Trabado J., Diebold Y., Sanchez A. (2017). Designing lipid NPs for topical ocular drug delivery. Int. J. Pharm..

[39] Khan N.R., Harun M.S., Nawaz A., Harjoh N., Wong T.W. (2015). Nanocarriers and their actions to improve skin permeability and transdermal drug delivery. Curr. Pharm. Des..

[40] Chu B., Zhang L., Qu Y., Chen X., Peng J., Huang Y., Qian Z. (2013). Synthesis characterization and drug loading property of Monomethoxy-Poly (ethylene glycol)-Poly (ε-caprolactone)-Poly (D, L-lactide) (MPEG-PCLA) copolymers. Sci. Rep..

[41] Olbrich C., Kayser O., Müller R.H. (2002). Lipase degradation of Dynasan 114 and 116 solid lipid NPs (SLN)—Effect of surfactants, storage time and crystallinity. Int. J. Pharm..

[42] Budhian A., Siegel S.J., Winey K.I. (2005). Production of haloperidol loaded PLGA NPs for extended controlled drug release of haloperidol. J. Microencapsul..

[43] Mohammed M.A., Syeda J., Wasan K.M., Wasan E.K. (2017). An overview of chitosan NPs and its application in non-parenteral drug delivery. Pharmaceutics.

[44] Üner M., Yener G. (2007). Importance of solid lipid NPs (SLN) in various administration routes and future perspectives. Int. J. Nanomed..

[45] Som I., Bhatia K., Yasir M. (2012). Status of surfactants as penetration enhancers in transdermal drug delivery. J. Pharm. Bioallied Sci..

[46] Khare A., Singh I., Pawar P., Grover K. (2016). Design and evaluation of voriconazole loaded solid lipid nanoparticles for ophthalmic application. J. Drug. Deliv..

[47] Pandey A., Mittal A., Chauhan N., Alam S. (2014). Role of surfactants as penetration enhancer in transdermal drug delivery system. J. Mol. Pharm. Org. Process. Res..

[48] Schmidberger M., Nikolic I., Pantelic I., Lunter D. (2019). Optimization of rheological behaviour and skin penetration of thermogelling emulsions with enhanced substantivity for potential application in treatment of chronic skin diseases. Pharmaceutics.

[49] Pajić N.B., Vucen S., Ilić T., O’Mahony C., Dobričić V., Savić S. (2021). Comparative efficacy evaluation of different penetration enhancement strategies for dermal delivery of poorly soluble drugs—A case with sertaconazole nitrate. Eur. Pharm. Sci..

[50] Akhtar N., Rehman M.U., Khan H.M.S., Rasool F., Saeed T., Murtaz G. (2011). Penetration enhancing effect of polysorbate 20 and 80 on the in vitro percutaneous absorption of lascorbic acid. Trop. J. Pharm. Res..

[51] D’souza A.A., Shegokar R. (2016). Polyethylene glycol (PEG): A versatile polymer for pharmaceutical applications. Expert Opin. Drug. Deliv..

[52] Fantini A., Demurtas A., Nicoli S., Padula C., Pescina S., Santi P. (2020). In vitro skin retention of crisaborole after topical application. Pharmaceutics.

[53] Sudhakar K., Fuloria S., Subramaniyan V., Sathasivam K.V., Azad A.K., Swain S.S., Sekar M., Karupiah S., Porwal O., Sahoo A. (2021). Ultraflexible Liposome Nanocargo as a Dermal and Transdermal Drug Delivery System. Nanomaterials.

[54] Uchechi O., Ogbonna J.D., Attama A.A. (2014). NPs for dermal and transdermal drug delivery. Application of Nanotechnology in Drug Delivery.

[55] Panapisal V., Charoensri S., Tantituvanont A. (2012). Formulation of microemulsion systems for dermal delivery of silymarin. AAPS PharmSciTech.

[56] Lopes L.B. (2014). Overcoming the cutaneous barrier with microemulsions. Pharmaceutics.

[57] Tian B., Yan Q., Wang J., Ding C., Sai S. (2017). Enhanced antifungal activity of voriconazole-loaded nanostructured lipid carriers against Candida albicans with a dimorphic switching model. Int. J. Nanomed..

